# A Novel Gonadotropin-Releasing Hormone 1 (*Gnrh1*) Enhancer-Derived Noncoding RNA Regulates *Gnrh1* Gene Expression in GnRH Neuronal Cell Models

**DOI:** 10.1371/journal.pone.0158597

**Published:** 2016-07-07

**Authors:** Polly P. Huang, Liza E. Brusman, Anita K. Iyer, Nicholas J. G. Webster, Pamela L. Mellon

**Affiliations:** 1 Department of Reproductive Medicine, University of California San Diego, La Jolla, California, United States of America; 2 Department of Medicine, University of California San Diego, La Jolla, California, United States of America; 3 Center for Reproductive Science and Medicine, University of California San Diego, La Jolla, California, United States of America; Florida International University, UNITED STATES

## Abstract

Gonadotropin-releasing hormone (GnRH), a neuropeptide released from a small population of neurons in the hypothalamus, is the central mediator of the hypothalamic-pituitary-gonadal axis, and is required for normal reproductive development and function. Evolutionarily conserved regulatory elements in the mouse, rat, and human *Gnrh1* gene include three enhancers and the proximal promoter, which confer *Gnrh1* gene expression specifically in GnRH neurons. In immortalized mouse hypothalamic GnRH (GT1-7) neurons, which show pulsatile GnRH release in culture, RNA sequencing and RT-qPCR revealed that expression of a novel long noncoding RNA at *Gnrh1* enhancer 1 correlates with high levels of GnRH mRNA expression. In GT1-7 neurons, which contain a transgene carrying 3 kb of the rat *Gnrh1* regulatory region, both the mouse and rat *Gnrh1* enhancer-derived noncoding RNAs (GnRH-E1 RNAs) are expressed. We investigated the characteristics and function of the endogenous mouse GnRH-E1 RNA. Strand-specific RT-PCR analysis of GnRH-E1 RNA in GT1-7 cells revealed GnRH-E1 RNAs that are transcribed in the sense and antisense directions from distinct 5’ start sites, are 3’ polyadenylated, and are over 2 kb in length. These RNAs are localized in the nucleus and have a half-life of over 8 hours. In GT1-7 neurons, siRNA knockdown of mouse GnRH-E1 RNA resulted in a significant decrease in the expression of the *Gnrh1* primary transcript and *Gnrh1* mRNA. Over-expression of either the sense or antisense mouse GnRH-E1 RNA in immature, migratory GnRH (GN11) neurons, which do not express either GnRH-E1 RNA or GnRH mRNA, induced the transcriptional activity of co-transfected rat *Gnrh1* gene regulatory elements, where the induction requires the presence of the rat *Gnrh1* promoter. Together, these data indicate that GnRH-E1 RNA is an inducer of *Gnrh1* gene expression. GnRH-E1 RNA may play an important role in the development and maturation of GnRH neurons.

## Introduction

Regulation of gonadotropin-releasing hormone (*Gnrh1*) gene expression and GnRH secretion are essential for normal reproductive maturation and fertility. During embryonic development, GnRH neurons originate in the olfactory placode then migrate to the developing forebrain. In adulthood, mature GnRH neurons are found scattered throughout the hypothalamus and form a small population of approximately 800 neurons that secrete GnRH decapeptide in a pulsatile fashion [1]. GnRH released from hypothalamic neurons stimulates the release of luteinizing hormone (LH) and follicle-stimulating hormone (FSH) from gonadotrope cells in the pituitary, and these glycoprotein hormones stimulate the gonads to produce sperm, oocytes, and steroid hormones. These precise mechanisms govern normal reproductive development and function, including puberty, menstrual cycle, pregnancy, and menopause.

Failure of GnRH neuron migration and maturation and dysregulation of *Gnrh1* gene expression have been implicated in the incorrect timing of puberty, reproductive deficiencies, and infertility. Reproductive disorders such as Kallmann Syndrome [2], Idiopathic Hypogonadotropic Hypogonadism [3], Prader-Willi Syndrome [4], and CHARGE Syndrome [5], are attributed to disruptions in *Gnrh1* gene expression, GnRH neuron signaling, and/or neuronal maturation. However, the majority of genetic causes and molecular mechanisms involved in the pathology of these reproductive deficiencies remain undefined. Thus, understanding the mechanisms regulating *Gnrh1* gene expression will provide important insights into the biology and pathophysiology of mammalian reproduction.

Immortalized, mature, mouse hypothalamic GnRH (GT1-7) neurons and immature, migratory GnRH (GN11) neurons serve as excellent cell culture models to study the unique and rare population of GnRH neurons in the hypothalamus. GT1-7 neurons were generated using a transgene containing 3 kb of rat *Gnrh1* 5’ regulatory region driving an oncogene (SV40 T-antigen) in mice [6] to produce tumors in the hypothalamus. GT1-7 neurons represent mature, differentiated GnRH neurons that express high levels of *Gnrh1* mRNA and secrete GnRH in a pulsatile manner [7–10]. GN11 cells were generated in mice using a transgene containing 1.1 kb of the human *GNRH1* 5’ regulatory region on SV40 T-antigen to produce a tumor in the nose [11]. GN11 cells represent immature, migratory GnRH neurons, which express very low levels of *Gnrh1* mRNA [12, 13].

Conserved regulatory elements in the mouse, rat, and human *Gnrh1* gene include three enhancers and the proximal promoter that, together, confer specific expression of *Gnrh1* in the discrete population of hypothalamic GnRH neurons. Three *Gnrh1* enhancers and the proximal promoter reside within 4.8 kb upstream of the mouse *Gnrh1* gene transcription start site (TSS) as defined by homology to their well-characterized counterparts in the rat gene. In the mouse gene, *Gnrh1* enhancer 3 (GnRH-E3) at -4688 bp/-4385 bp, enhancer 2 (GnRH-E2) at -3622 bp/-3100 bp, and enhancer 1 (GnRH-E1) at -2404 bp/-2100 bp facilitate transcriptional activity of the *Gnrh1* promoter (GnRH-P) at -278 bp/97 bp. Importantly, as shown in the rat gene by transfection into the mouse GT1-7 cell line, GnRH-E1 interacts with GnRH-E2, GnRH-E3 and GnRH-P in specifying robust GnRH neuron-specific gene expression, and lack activity in GN11 cells and NIH3T3 mouse fibroblasts [14–16].

The essential role of GnRH-E1 in mature GnRH neuron-specific *Gnrh1* transcriptional activity is also observed *in vivo*. Transgenic mice carrying rat GnRH-E1 and GnRH-P driving β-galactosidase showed GnRH neuron-specific expression, while rat GnRH-E1 on a heterologous promoter showed non-specific β-galactosidase expression in the brain [17]. Knock-out mice carrying a deletion in the endogenous mouse GnRH-E1 showed decreased *Gnrh1* mRNA expression in the brain, but showed higher ovarian *Gnrh1* mRNA expression accompanied by irregular estrous cyclicity [18]. These reports highlight the functional significance of GnRH-E1 and the relationship with *Gnrh1* promoter in the specificity of *Gnrh1* gene expression.

Transcription at enhancers has been described to be a genome-wide phenomenon in a constitutively active and regulated manner. Actively transcribed enhancers display signature histone marks of high H3K4me1 and low H3K4me3 [19], open chromatin structure, and RNA polymerase II (RNA Pol II) enrichment [20, 21]. Transcription at enhancers can generate functional noncoding RNAs with different transcriptional modalities, structures, and functions. Long noncoding RNAs (lncRNA) are defined as 5’ capped and 3’ polyadenylated RNA molecules of over 200 nt in length. LncRNAs are predominately spliced and are transcribed in one direction. LncRNA transcription can overlap intergenic regions, introns or exons of protein-coding genes [22], thus lncRNA transcription can overlap enhancers. On the other hand, transcription at enhancers can produce enhancer RNAs (eRNAs), which are categorized as a sub-class of lncRNAs. Transcription of eRNAs is generally bi-directional, starting from a central region, producing relatively short (0.5 kb– 2 kb) noncoding transcripts that predominantly lack polyadenylation or splicing [23]. While RNA transcription at enhancers has been widely observed as a pervasive phenomenon in the genome, the functional significance of the majority of enhancer-derived noncoding RNAs remain to be elucidated.

The initial discovery of *Gnrh1* enhancer-transcribed RNA highlighted the characteristics of actively transcribed enhancers. The endogenous mouse *Gnrh1* regulatory region displays DNase1 hypersensitivity, histone markers of actively transcribed enhancers (i.e. high H3K4me1 and low H3K4me3), and RNA pol II enrichment primarily in GT1-7 neurons, whereas histone markers of inactive chromatin were observed in GN11 and NIH3T3 cells as assayed by chromatin immunoprecipitation (ChIP). Treatment of GT1-7 cells with 12-O-tetradecanoyl-phorbol-13-acetate (TPA), an activator of the protein kinase C (PKC) pathway, repressed *Gnrh1* mRNA expression and reduced RNA Poll II occupancy at GnRH-E1. RT-PCR analyses showed the expression of *Gnrh1* enhancer-transcribed RNA robustly correlated with *Gnrh1* mRNA expression in response to PKC pathway signaling after TPA treatment only in GT1-7 neurons. [24]. These initial observations suggest functional significance of a novel noncoding RNA transcribed from an active enhancer in cell type-specific manner. In this study, we characterized the expression profile and function of the mouse *Gnrh1* enhancer-derived noncoding RNA (GnRH-E1 RNA) in GnRH model cell lines. We established GnRH-E1 RNA as a novel regulator of *Gnrh1* gene expression. Our data provide the foundation for future studies in the molecular mechanisms of *Gnrh1* regulation with implications in mammalian reproductive development and fertility.

## Materials and Methods

### Cell Culture, Actinomycin D Treatment and Cell Fractionation

GT1-7, GN11 (courtesy of Dr. Sally Radovick, Rutgers University, and Susan Wray, National Institutes of Health), and NIH3T3 cell lines were cultured in DMEM (Gemini Bio Products, West Sacramento, CA) with 4.5% glucose, 10% fetal bovine serum and 1% penicillin-streptomycin cocktail in 5% CO_2_ at 37°C. Cell lines at passage 15–25 were used in this study. For actinomycin D treatments, GT1-7 cells were seeded in 10 cm plates (Nunc, Denmark) at 300,000 cells/mL 24 hours prior to treatment. Cells were treated with actinomycin D or DMSO vehicle at 1 μg/mL final concentration. At the time points indicated, cells were rinsed with phosphate-buffered saline (PBS) and homogenized in Trizol Reagent (Thermo Fisher Scientific, Carlsbad, CA) for RNA extraction.

To harvest nuclear and cytoplasmic extracts from GT1-7 neurons in culture, cells were first aspirated and washed with PBS, followed by centrifugation at 1000 g for 5 minutes. The cell pellet was resuspended in cytoplasmic extract buffer (10 mM HEPES, 60 mM KCl, 1 mM EDTA, 0.075% NP40, 1 mM DTT, and 1 mM PMSF) on ice for 3 minutes. After centrifugation at 1500 g for 4 minutes, the cytoplasmic extract (supernatant) was homogenized in Trizol reagent for RNA extraction. The pellet containing nuclei was resuspended in cytoplasmic extract buffer without NP40. After centrifugation of the nuclei at 1500 g for 4 minutes, the buffer was removed and the nuclei were resuspended in nuclear extract buffer (20 mM Tris at pH 7.9, 10 mM NaCl, 1.5 mM MgCl_2_, 0.2 mM EDTA, and 1 mM PMSF) and incubated on ice for 10 minutes. After centrifugation at 13,000 g for 10 minutes, the nuclear extract buffer was removed and the pellet containing nuclei was homogenized in Trizol reagent for RNA extraction.

### Reverse Transcription-PCR and Quantitative PCR

Total RNA was harvested from GT1-7, GN11, and NIH3T3 cells using Trizol Reagent according to the manufacturer’s recommendations. Genomic DNA was removed from RNA samples using TURBO DNA-free kit (Thermo Fisher Scientific, Carlsbad, CA) according to the manufacturer’s recommendations. First-strand cDNA was synthesized from the total RNA from each cell line, using SuperScript III First-Strand cDNA Synthesis System (Thermo Fisher Scientific, Carlsbad, CA), according to the manufacturer’s recommendations, and was primed by either oligo(dT)_20_ primers (from cDNA synthesis kit) or gene-specific primers (IDT DNA Technologies, San Diego, CA), where indicated. Gene-specific primers for cDNA synthesis are listed in Table 1.

**Table 1:** Oligonucleotide sequences.

Primer Name	*	Sequence (5’ to 3’)	Figures
Mouse GnRH-E1 RNA -3371	F	GGGAAAGGAAAGCAATTTCA	3C-D
Mouse GnRH-E1 RNA -3560	F	AGCTGTGTCCAAATGGGTTC	3A-D; 7A and D
Mouse GnRH-E1 RNA -3606	F	CGTAGGTGTCCCAGTGTCCTTATTTGTTGC	3A-B
Mouse GnRH-E1 RNA -3746	F	CTCTATGTCAGTACCTGTGGCTCGGGCTCG	3A-B
Mouse GnRH-E1 RNA -3779	F	TGAACAAAGGAAGCATTTGG	3A-B
Mouse GnRH-E1 RNA -1271	R	GCAGATGCTGCCTCTATTTCA	3A-D
Mouse GnRH-E1 RNA -1128	R	GCCTGCCTCTGAAACTTTTG	3A-D
Mouse GnRH-E1 RNA -1443	R	TGCTAACCCCAATCCGCCATTGCTT	3C-D
Mouse GnRH-E1 RNA -2438	R	GCGTCAAACTACAAAGGAAAGTGGCTGCAT	2A and C; 5A; 6A
Mouse GnRH-E1 RNA -2106	R	TAGGCCAATTTACCCAAGACCTGAAATTGA	2A and C; 5A; 6A
Rat GnRH-E1 RNA -1640	F	GCAGACAGAGGAGCTGAGAGATGCAAAC	2B and C; 5B; 6B
Rat GnRH-E1 RNA -1385	R	AGGGTAGGGAAGAGGGACTTTGTGGTG	2B and C; 5B; 6B
Mouse *Gnrh1* mRNA +1144 (exon)	F	CTACTGCTGACTGTGTGTTTG	5C-D; 6C-D
Mouse *Gnrh1* mRNA +4059 (exon)	R	CATCTTCTTCTGCCTGGCTTC	5C; 6C
*Gnrh1* pre-mRNA +1297 (intron)	R	GCTCATGCAGTTTGAAGCGAGTC	5D; 6D
Mouse *H2afz* mRNA	F	TCACCGCAGAGGTACTTGAG	5 and 6
Mouse *H2afz* mRNA	R	GATGTGTGGGATGACACCA	
Mouse *Ppia* mRNA	F	AAGTTCCAAAGACAGCAGAAAAC	2C
Mouse *Ppia* mRNA	R	CTCAAATTTCTCTCCGTAGATGG	2C
Mouse GnRH-E1 RNA siRNA sense		AAACUACAAAGGAAAGUGG	6
Mouse GnRH-E1 RNA siRNA antisense		CCACUUUCCUUUGUAGUUU	6
siRNA negative control pool		UGGUUUACAUGUCGACUAA, UGGUUUACAUGUUGUGUGA, UGGUUUACAUGUUUUCUGA, UGGUUUACAUGUUUUCCUA	6

* Indicates primer direction of forward (F) or reverse (R).

PCR was performed using FastStart *Taq* DNA Polymerase (Roche Diagnostics, Indianapolis, IN) and the following conditions: 95°C for 5 minutes denature, followed by 30 cycles of 95°C for 45 seconds, 60°C for 45 seconds, 72°C for 45 seconds, and final extension of 72°C for 10 minutes. PCR primers are listed in Table 1 and were tested for optimal annealing temperatures and PCR conditions. RT-PCR products were labeled by the addition of 10 μg/mL of ethidium bromide and were resolved on a 1% agarose gel for >1 kb PCR product and 2% agarose gel for ≤ 500 bp PCR product in 1X TAE buffer (40 mM Tris at pH 7.6, 20 mM acetic acid and 1 mM EDTA).

Quantitative PCR was performed using iQ SYBR Green Supermix and iQ5 Real-Time PCR detection system and software (Bio-Rad, Hercules, CA) according to the manufacturer’s recommendations. Standard curves were generated using serial dilutions of plasmid DNA containing the qPCR amplicon cloned into pcDNA2.1 backbone. Quantities of GnRH-E1 RNA, *Gnrh1* pre-mRNA, and *Gnrh1* mRNA were normalized to histone 2A.Z (*H2afz*) mRNA control or peptidylprolyl isomerase A (*Ppia*) mRNA control, where indicated. RT-qPCR primers are listed in Table 1. PCR and qPCR primers for GnRH mRNA have been previously described [25].

### RNA-Sequencing

RNA was isolated from GT1-7, GN11, and NIH3T3 cells using TRIzol® (Invitrogen, Thermo Fisher Scientific, Carlsbad, CA), as per the manufacturer’s instructions, and treated with Turbo DNA-free DNase (Ambion, Thermo Fisher Scientific, Carlsbad, CA). Concentration was determined using SmartSpec Plus Spectrophotometer System (BioRad, Irvine, CA). The RNA integrity (RNA Integrity Number ≥ 9) and quantity was determined on the Agilent 2100 Bioanalyzer (Agilent, Palo Alto, CA, USA). cDNA libraries were created using the TruSeq™ RNA Sample Prep-v2 (Illumina, San Diego, CA), using the manufacturer’s low-throughput protocol. Indexed samples were mixed at equal concentrations, four samples per lane, and sequenced using the HiSeq 2000 sequencer (Illumina, San Diego, CA). The resulting Fastq sequence reads were validated and analyzed in Galaxy [26] using FASTQC (www.bioinformatics.babraham.ac.uk/projects/fastqc/) and FASTQ Groomer [27], and any remaining adapter sequences were removed using FASTQ Clipper program (http://hannonlab.cshl.edu/fastx_toolkit/index.html). The reads were aligned to the mouse genome (mm10 assembly) using the TopHat2 program [28] and differential expression was analyzed using Cufflinks software program [29]. Sequence read alignments (accepted-hit bam files) were visualized using the Integrative Genome Viewer [30, 31]. Sequencing library preps, RT-qPCR, sequencing, and alignment were performed by the UCSD BIOGEM Core facility supported by NIH grants P30 DK063491 and P30 CA023100. Primary RNA sequencing reads have been deposited to the NIH Short Read Archive: Accession number SRP075629.

### Plasmids

The -5 kb rat *Gnrh1*-luciferase reporter and 5’ truncations were generated as previously described [14]. The rat GnRH-E1/GnRH-P luciferase reporter in pGL3 vector have been previously described [32, 33]. The luciferase reporters carrying the Rous sarcoma virus (RSV) long terminal repeat enhancer and promoter RSVe/RSVp, GnRH-E1/RSVp, RSVe/GnRH-P, and GnRH-E1/RSVp on pGL3 vector have been previously described [15]. The mouse GnRH-E1 RNA expression plasmid was constructed by PCR amplification of the 2432 bp (-3560 bp/-1128 bp) segment from GT1-7 neuron genomic DNA using Platinum Pfx DNA polymerase kit (Thermo Fisher Scientific, Carlsbad, CA). The segment was inserted at the *EcoR1* restriction enzyme digest site of the pcDNA 2.1 (Invitrogen, Thermo Fisher Scientific, Carlsbad, CA) backbone plasmid using T4 DNA ligase (New England Biolabs, Ipswich, MA). Plasmid constructs carrying -3560 bp/-1128 bp of GnRH-E1 RNA, integrated in the forward or the reverse orientation, were verified by DNA sequencing. For quantitative PCR of the transgene-transcribed rat GnRH-E1 RNA, the standard plasmid was constructed by cloning -2854 bp/-1156 bp segment of the rat transgene from GT1-7 neuron genomic DNA into the *EcoR1* restriction enzyme digest site of the pcDNA 2.1 backbone plasmid.

### Transient Transfections and Reporter Assays

For luciferase assays, GT1-7 and GN11 cells were seeded into 24-well plates 24 hours before transfection at concentrations of 250,000 cells/mL and 70,000 cells/mL, respectively. Plasmids were transfected using PolyJet^TM^ Transfection Reagent (SignaGen Laboratories, Rockville, MD) according to the manufacturer’s recommendations. Cells were co-transfected with 150 ng/well of luciferase reporter plasmid, 200 ng/well of expression plasmid, and 100 ng/well of thymidine kinase β-galactosidase reporter plasmid as internal control for transfection efficiency. Cells were transfected in parallel with RSVp-Luciferase or pGL3-Luciferase reporter plasmid where indicated. At 8 hours after transfection, cells were serum starved (DMEM with 0.1% bovine serum albumin and 1% penicillin/streptomycin cocktail) for 24 hours prior to harvest. Cells were harvested using cold lysis buffer (100 mM potassium phosphate at pH 7.8 and 0.2% Triton X-100). Luciferase and β-galactosidase assays were performed using the Galacto-Light Plus System (Thermo Fisher Scientific, Carlsbad, CA). Three independent experiments were done, each in triplicate.

For siRNA transfections, GT1-7 cells were seeded in 6-well plates at the concentration of 250,000 cells/mL 48 hours prior to transfection. Growth media was replaced with DMEM with 4.5% glucose and 5% fetal bovine serum 24 hours prior to siRNA transfection. Negative siRNA control targeting luciferase mRNA and custom-designed siRNA duplex oligos targeting GnRH-E1 RNA (Invitrogen, Thermo Fisher Scientific, Carlsbad, CA) and Lipofectamine RNAiMax transfection reagent (Thermo Fisher Scientific, Carlsbad, CA) were prepared according to the manufacturer’s recommendations, in Gibco OptiMEM (Thermo Fisher Scientific, Carlsbad, CA) at a concentration of 10 μM. siRNA was transfected into GT1-7 cells at a concentration of 900 pmol per well. Three independent experiments were performed, each in triplicate.

### Data Analysis

All transient transfections, RT-PCR and quantitative RT-PCR were independently repeated three times or more. For transient transfections, luciferase activity was normalized relative to β-galactosidase to control for transfection efficiency. Unless otherwise noted, the data were normalized to luciferase:β-galactosidase activity ratio of empty pGL3 plasmid transfected in parallel. The data were compared between cells transfected with empty pcDNA2.1 plasmid and those transfected with pcDNA2.1 plasmid carrying GnRH-E1 RNA. Results are presented as mean ± standard deviation (SD) of the fold induction relative to pGL3. Statistical analyses were performed on luciferase activity normalized to β-galactosidase. For RT-qPCR, the mean copy number for GnRH-E1 RNA, *Gnrh1* pre-mRNA and *Gnrh1* mRNA were normalized to *H2afz* mRNA copy number as internal control. Student’s *t*-test and 2-way ANOVA followed by Tukey-Kramer HSD *post hoc* tests were used as indicated, where P<0.05 indicated statistical significance.

## Results

### Expression of GnRH-E1 RNA in Mouse Immortalized Hypothalamic GnRH Neurons

**Fig 1:**
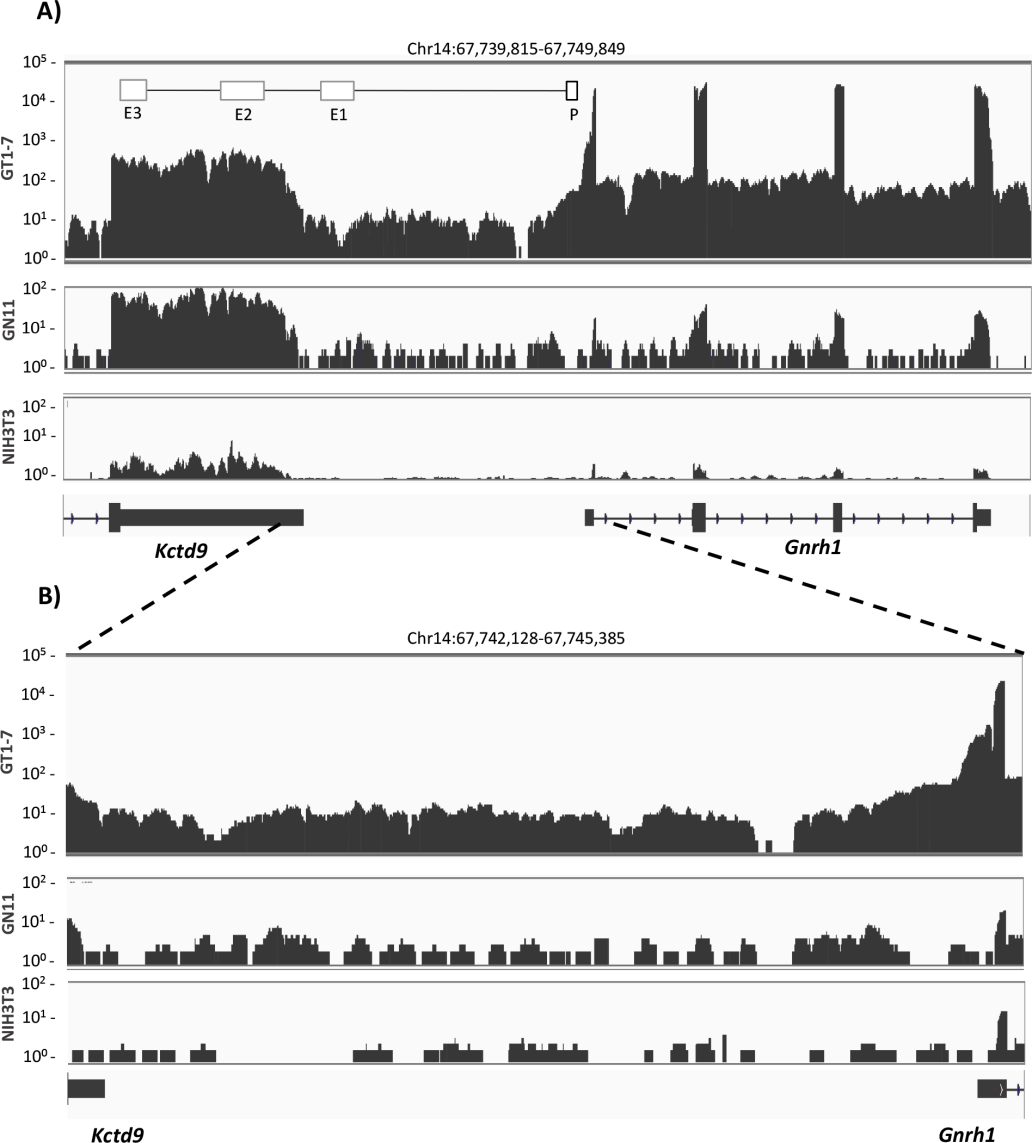
RNA sequencing analysis of the upstream regulatory region of the *Gnrh1* gene. (A-B) RNA expression in GnRH model cell lines GN11 and GT1-7, and NIH3T3 mouse fibroblasts are displayed in tracks as labeled. The tracks show the number of sequence reads on the y-axis, aligned to chromosome location on the x-axis. The RNA reads are displayed in log scale: 1–10^5^ for GT1-7 cells, and 1–10^2^ for GN11 and NIH3T3 cells. (A) RNA sequencing analysis of the mouse *Gnrh1* gene and the upstream intergenic region between *Kctd9* and *Gnrh1* is shown. RNA reads are aligned to *Gnrh1*, the upstream intergenic region, and the 3’ untranslated region (UTR) of *Kctd9* (mouse Chr14:67,739,815–67,749,849 of the mouse mm10 genome assembly). A schematic diagram of the *Gnrh1* enhancers (E1, E2, E3) and promoter (P) are shown in the GT1-7 track, and are aligned to the 3’ UTR of *Kctd9* and the genomic region that is 5’ of *Gnrh1*. (B) An enlarged display of RNA reads aligned to the intergenic region located upstream of *Gnrh1* and the 3’ UTR of *Kctd9* (mouse Chr14: 67,742,182–67,745,385).

We first examined GnRH-E1 RNA expression using RNA sequencing analysis of model cell lines. The mouse *Gnrh1* gene is located on mouse chromosome 14 and is flanked by the genes *Kctd9* transcribed in the same direction as *Gnrh1* and located upstream of *Gnrh1*, and *Dock5* transcribed in the opposite direction and located downstream of *Gnrh1*. Mouse *Gnrh1* enhancers and promoter are located in the genomic region 5’ of *Gnrh1*, where *Gnrh1* enhancers 2 and 3 (E2 and E3) are located in the region overlapping the 3’ UTR of *Kctd9*, and *Gnrh1* enhancer 1 (E1) and the promoter are located in the intergenic region 3’ of *Kctd9*. The robust detection of RNA reads that align to the exons of the *Gnrh1* gene confirms the high level of *Gnrh1* expression in GT1-7 cells (Fig 1A). In contrast, GN11 cells show fewer exonic RNA reads, and *Gnrh1* is virtually not expressed in NIH3T3 fibroblasts (note the different log scale for GT1-7, compared to GN11 and NIH3T3). RNA reads were also detected that align to the introns of the *Gnrh1* gene and likely represent the signal from the *Gnrh1* primary transcript, as the sequencing analysis was performed on total RNA. In addition to the reads aligning to *Gnrh1*, GT1-7 cells showed dense and robust RNA reads that align to the intergenic region of *Gnrh1* and *Kctd9* genes (Fig 1B). The intergenic RNA reads were indistinguishable from background in GN11 and NIH3T3 cells. Together, these observations are consistent with our previous report of the positive correlation between RNA expression in the upstream regulatory region of the *Gnrh1* gene and GT1-7 cell-specific *Gnrh1* mRNA expression using quantitative PCR analysis [24].

**Fig 2:**
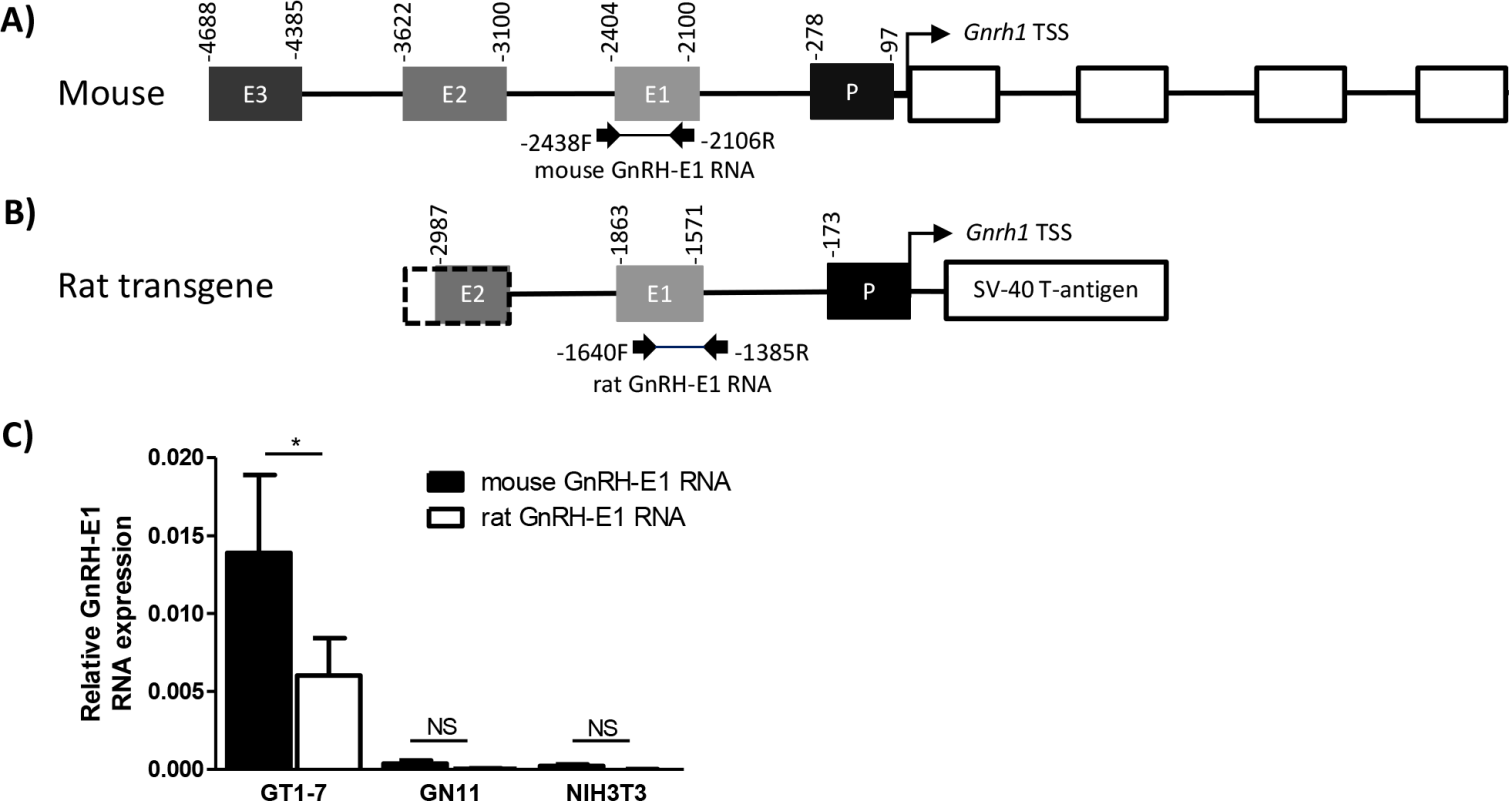
RT-qPCR analysis of rat and mouse GnRH-E1 RNA expression in cell lines. (A) A schematic diagram of conserved regulatory elements upstream of the mouse *Gnrh1* transcription start site (TSS, curved arrow) and *Gnrh1* gene coding region. The *Gnrh1* gene regulatory region contains *Gnrh1* enhancers 1, 2, and 3 (E3, E2, E1, respectively), the promoter (P), and the *Gnrh1* gene consists of four exons (white boxes). PCR primers used in Fig 2C are indicated by arrows, and the predicted PCR product mouse GnRH-E1 RNA is represented by a connecting line. (B) Schematic diagram of transgene embedded in GT1-7 neurons carrying the 3’ portion of the rat GnRH-E2, GnRH-E1, GnRH-P, and the *Gnrh1* TSS, driving the SV40 T-antigen oncogene. PCR primers used in Fig 2C are indicated by arrows, and the predicted PCR product rat GnRH-E1 RNA from the transgene is represented by a connecting line. (C) RT-qPCR analysis of endogenous mouse GnRH-E1 RNA (black) and GnRH-E1 RNA expressed from the rat *Gnrh1* promoter transgene (white) in GT1-7, GN11, and NIH3T3 cells. Relative GnRH-E1 RNA expression is normalized to peptidylprolyl isomerase A (*Ppia*) mRNA control. Data are displayed as means ± SD. Asterisk indicates statistical significance by Student’s T-test on the comparison between mouse and rat GnRH-E1 RNA, where p<0.05.

The mouse *Gnrh1* gene regulatory region consists of three enhancers and the promoter located upstream of the *Gnrh1* transcription start site (Fig 2A). GT1-7 neurons also carry a transgene that consists of 3.0 kb of the rat *Gnrh1* gene regulatory region (Fig 2B). RT-qPCR analysis of oligo-dT-primed cDNA from GnRH cell models revealed that endogenous RNA expression from the mouse *Gnrh1* regulatory region is significantly higher than RNA expression from the rat transgene in GT1-7 neurons (Fig 2C). The data indicate that the rat and mouse GnRH-E1 RNAs are polyadenylated. We continued to characterize the expression and function of the endogenous mouse GnRH-E1 RNA in mouse GnRH model cell lines.

We examined the structure of the endogenous mouse GnRH-E1 RNA. Preliminary analysis using rapid amplification of cDNA ends (RACE) indicated alternate 5’ capping structure and 3’ polyadenylation sites located upstream and downstream of GnRH-E1, which suggests the expression of GnRH-E1 RNA variants transcribed from the sense and antisense direction in GT1-7 cells. However, alternate locations of 5’ capping structure were identified at approximately -3458 bp and -1576 bp, and 3’ polyadenylation sites at approximately -3720 bp and at -1182 bp from the *Gnrh1* TSS were revealed by our preliminary analysis by RACE. Conventional RT-PCR analysis using oligo-dT-primed cDNA consistently produced full-length amplicons of over 2 kb in length, extending across GnRH-E1. To overcome the limitations of RACE, confirm the 5’ and 3’ ends of GnRH-E1 RNA variants, as well as to confirm the sense and antisense transcription of GnRH-E1 RNA, we used the alternative approach of strand-specific RT-PCR. We synthesized strand-specific cDNA from GT1-7 total RNA using gene-specific primers for cDNA synthesis and RT-PCR analysis.

**Fig 3:**
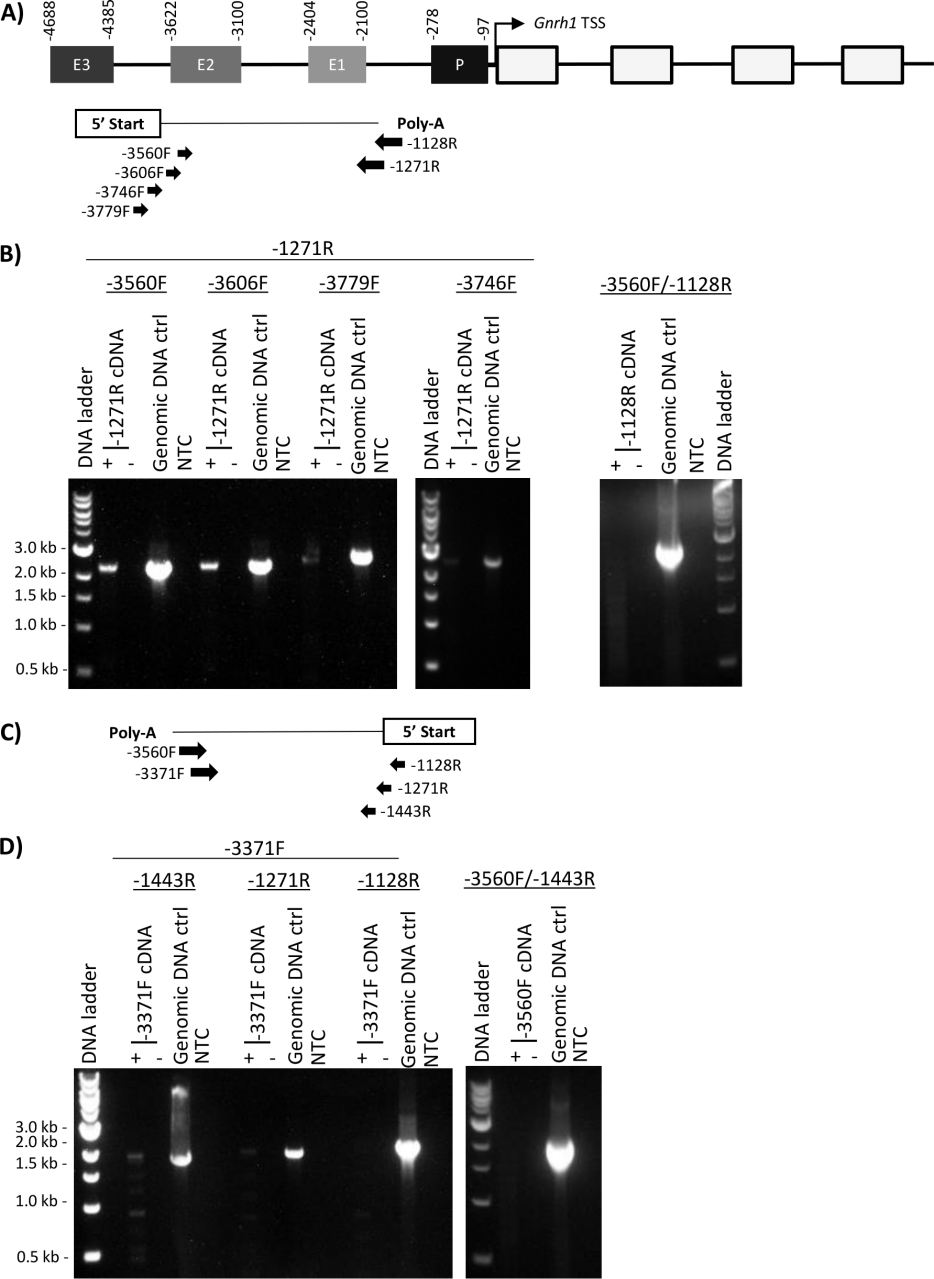
Strand-specific cDNA and RT-PCR analysis of the mouse GnRH-E1 RNA variants. (A) Schematic diagram of the sense GnRH-E1 RNA variant, with a 3’ polyA site located downstream of GnRH-E1 as predicted by RACE. PCR primers in reverse direction at -1128 bp or -1271 bp (reverse arrows) were used for strand-specific cDNA synthesis to capture the sense GnRH-E1 RNA. PCR analysis was performed using -1271 bp or -1128 bp reverse primer paired with the forward primers at -3560 bp, -3606 bp, -3779 bp, and -3746 bp (forward arrows) from the mouse *Gnrh1* TSS. Primer positions are aligned to the mouse conserved regulatory elements and coordinates diagrammed above. (B) Strand-specific cDNA synthesized using the -1271 bp reverse primer was subject to PCR analysis using the following primer pairs: -3560F/-1271R, -3606F/-1271R, -3779F/-1271R, -3746F/-1271R. Strand-specific cDNA synthesized using -1128 bp reverse primer was subject to PCR analysis using the primer pair -3560F/-1128R. (C) Schematic diagram of the mouse antisense GnRH-E1 RNA variant, with a 3’ polyA site predicted upstream of GnRH-E2 by RACE. PCR primers in the forward direction at -3560 bp or -3371 bp (forward arrows) was used for strand-specific cDNA synthesis to capture the antisense GnRH-E1 RNA variant. PCR analysis was performed using -3371 bp or -3560 bp forward primer paired with the reverse primers at -1443 bp, -1271 bp, -1128 bp from the *Gnrh1* TSS. D) Strand-specific cDNA synthesized using the -3371 bp forward primer was subject to PCR analysis using the following PCR primer pairs -3371F/-1443R, -3371F/-1271R, and -3371F/-1128R. Strand-specific cDNA synthesized using the -3560 bp forward primer was subject to PCR analysis using the primer pair at -3560F/-1443R. All reverse transcription reactions were performed on total RNA samples with (+) and without (-) reverse transcriptase and were amplified by PCR in parallel with GT1-7 genomic DNA control and no-template water control (NTC). The size of PCR amplicons was marked by 1 kbp DNA ladder.

To capture the mouse sense GnRH-E1 RNA variant, we used reverse primers at -1128 bp or -1271 bp for first-strand cDNA synthesis. The cDNA synthesized using the gene-specific primer (GSP) at -1271 bp (reverse) was subjected to RT-PCR analysis using the same reverse primer, paired with forward primers at -3560 bp, -3606 bp, -3746 bp, and -3779 bp (Fig 3A). PCR products of -3560 bp/-1271 bp and -3606 bp/-1271 bp were successfully detected, but PCR products from -3746 bp/-1271 bp and -3779 bp/-1271 bp were greatly diminished (Fig 3B). When the cDNA synthesized from using the gene-specific primer at -1128 bp (reverse) was subjected to RT-PCR analysis using the same reverse primer, paired with the forward primer at -3560 bp, no PCR product was observed. Reverse transcriptase reaction using GSP -1128 bp (reverse) failed to capture the 3’ end of the sense GnRH-E1 RNA variant. Together, the data suggest that the transcription start site of the sense GnRH-E1 RNA variant resides between -3606 bp and -3746 bp, and that a 3’ polyA termination site is located between -1271 bp and -1128 bp from the *Gnrh1* TSS.

Similarly, to capture the mouse antisense GnRH-E1 RNA variant, we used forward primers at -3560 bp and at -3371 bp for first-strand cDNA synthesis. The cDNA synthesized using the gene-specific primer at -3371 bp (forward) was subject to RT-PCR analysis using the same forward primer, paired with reverse primers at -1128 bp, -1271 bp, and -1443 bp (Fig 3C). PCR products of -3371 bp/-1443 bp and -3371 bp/-1271 bp were successfully detected, though the PCR product of -3371 bp/-1271 bp was greatly diminished. The expected PCR product from -3371 bp/-1128 bp was not observed. When the cDNA synthesized using the GSP at -3560 bp (forward) was subject to PCR analysis using the same forward primer, paired with the reverse primer at -1443 bp, no PCR product was observed (Fig 3D). PCR products of smaller size were resolved on the agarose gel, along with PCR products of -3371 bp/-1443 and -3371 bp/-1271 bp, but DNA sequencing of the shorter PCR products revealed sequences that did not align to the upstream region of *Gnrh1* gene and suggesting non-specific PCR amplification. First-strand cDNA synthesis using GSP -3560 bp forward failed to capture the 3’ end of the antisense GnRH-E1 RNA variant. Together, the data indicate that the TSS of the antisense GnRH-E1 RNA variant likely resides between -1443 bp and -1271 bp, and that a polyA termination site is located between -3560 bp and -3371 bp from the *Gnrh1* TSS.

Our strand-specific RT-PCR analyses of GnRH-E1 RNA indicate that sense and antisense variants are over 2 kb in length. We did not observe shorter PCR products within the -3746 bp to -1128 bp region, which suggests that GnRH-E1 RNA variants are not spliced. Analysis by Coding Potential Calculator [34] revealed that the -3746 bp/-1128 bp segment, and its reverse complementary strand, of the mouse *Gnrh1* gene do not have coding potential. Furthermore, our data indicate that the sense GnRH-E1 RNA is transcribed from a distinct 5’ start site located upstream of GnRH-E1, and the antisense GnRH-E1 RNA is transcribed from a start site located downstream of GnRH-E1. The 5’ transcription start of sense and antisense GnRH-E1 RNA variants do not originate from a central transcription start site. In addition, our approximated boundaries of the sense and antisense GnRH-E1 RNAs between -1271 bp and -1128 bp from the *Gnrh1* TSS coincides with a decrease in the amount of RNA reads at approximately -1168 bp from the *Gnrh1* TSS. Together, our data indicate that GnRH-E1 RNA variants are likely 5’ capped, 3’ polyadenylated RNA molecules of over 2 kb in length, and do not contain functional open reading frames (ORFs). These characteristics and the location of GnRH-E1 RNA transcription are consistent with the features of lincRNAs, a sub-category of lncRNAs transcribed from intergenic regions including enhancers.

### GnRH-E1 RNA Is a Stable Long Intergenic Noncoding RNA (lincRNA) Localized in the Nucleus

LncRNAs are defined as RNA molecules of more than 200 nucleotides in length with low coding potential, but share similar features of protein-coding RNAs, including 5’ capping structure, splicing, and 3’ polyadenylation [22]. LncRNAs have been observed to localize in the nucleus and/or in the cytoplasm, with specialized functional roles. LncRNAs localized in the cytoplasm can participate in the regulation of mRNA translation, while lncRNAs in the nucleus provide structural support and regulate gene transcription [35]. Furthermore, lncRNAs function to regulate target gene transcription via a variety of mechanisms, such as interacting with chromatin modifiers, as DNA-binding complexes, regulators of post-transcriptional processing, to sequester microRNAs, or as structural scaffold in nuclear domains [36]. The expression level of bi-directionally transcribed eRNAs can be positively correlated with transcription level of proximal genes, and eRNAs have been described to participate in facilitating enhancer-promoter DNA looping interactions [37, 38].

**Fig 4:**
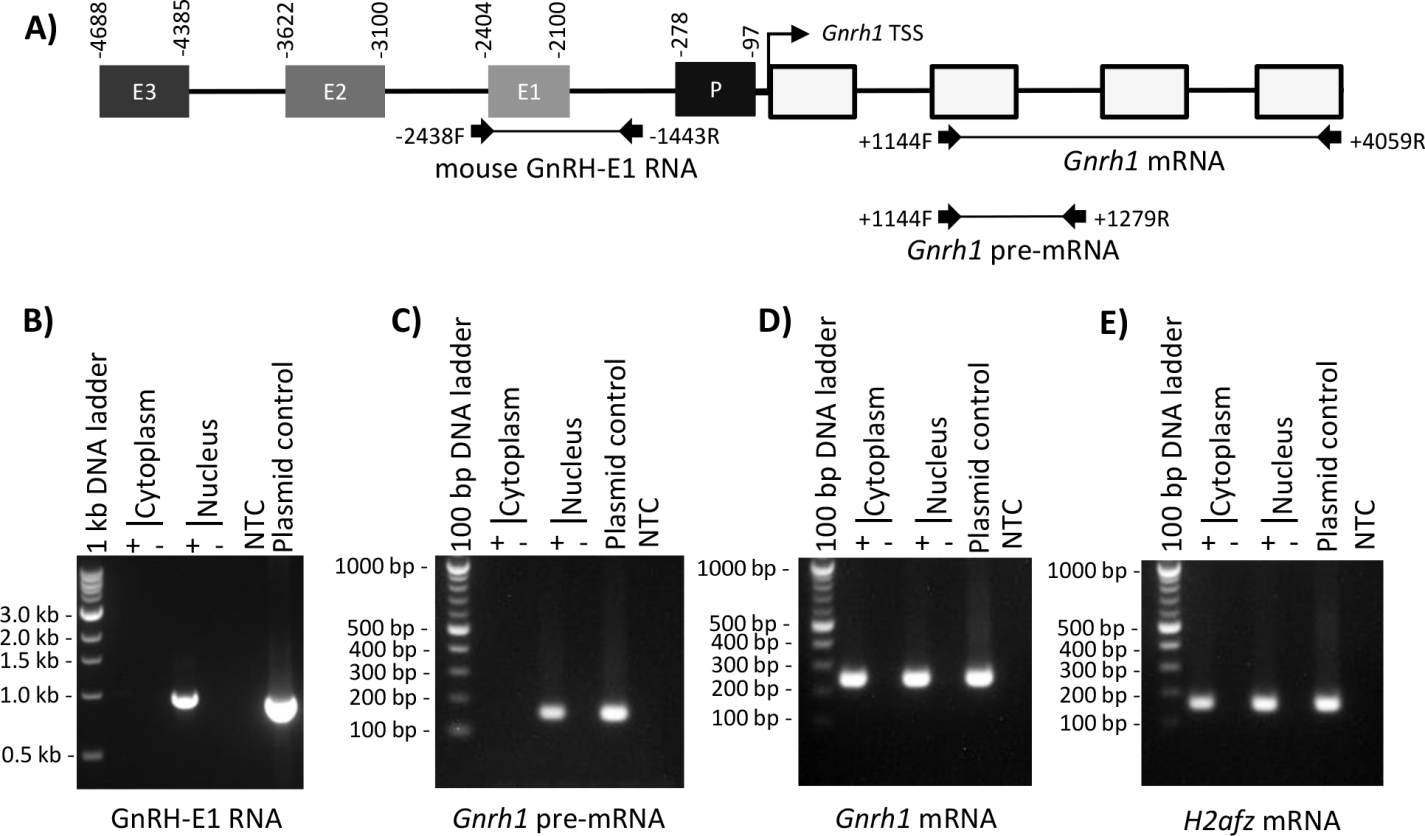
GnRH-E1 RNA is localized in the GT1-7 neuron nucleus. (A) Schematic diagram of the conserved regulatory elements upstream of the mouse *Gnrh1* TSS, which contains enhancers 1, 2, and 3 (E3, E2, E1, respectively), the promoter (P), and the *Gnrh1* gene with four exons (white boxes). Coordinates above the regulatory elements indicate positions with respect to the *Gnrh1* TSS. RT-PCR primers used in B-D are indicated by arrows, and expected PCR products are represented by a connecting line. Positions of PCR primers are aligned to the mouse conserved regulatory region diagrammed above. Nuclear and cytoplasmic extracts from GT1-7 neurons were analyzed for GnRH-E1 RNA (B), *Gnrh1* pre-mRNA (C), *Gnrh1* mRNA (D), and *H2afz* mRNA control (E) by RT-PCR. RT-PCR analysis was performed on random hexamer-primed cDNA, where cDNA synthesized with (+) and without (-) reverse transcriptase were analyzed in parallel. PCR loading controls are plasmid containing the -3568/-1128 bp segment upstream of the *Gnrh1* TSS and no-template control (NTC). The sizes of the PCR amplicons were marked by a 100 bp DNA ladder or a 1 kbp DNA ladder where indicated, that were resolved on the agarose gel in parallel.

We studied the localization and stability of GnRH-E1 RNA to gain additional insight into the functional role in *Gnrh1* gene regulation. RT-PCR analysis of nuclear and cytoplasmic extracts from GT1-7 neurons revealed that GnRH-E1 RNA is present in the nuclear RNA and absent from the cytoplasmic RNA. Appropriately, *Gnrh1* primary transcript (pre-mRNA) was observed in the nucleus, whereas *Gnrh1* mRNA was observed in both the nuclear and cytoplasmic extracts (Fig 4). The localization of *Gnrh1* mRNA in both the nucleus and cytoplasm is consistent with the robust *Gnrh1* expression and GnRH synthesis in GT1-7 neurons. The predominantly nuclear localization of GnRH-E1 RNA suggests functional roles in the nucleus, such as the regulation of gene expression at the level of transcription, rather than the participation in mRNA translation control in the cytoplasm.

**Fig 5:**
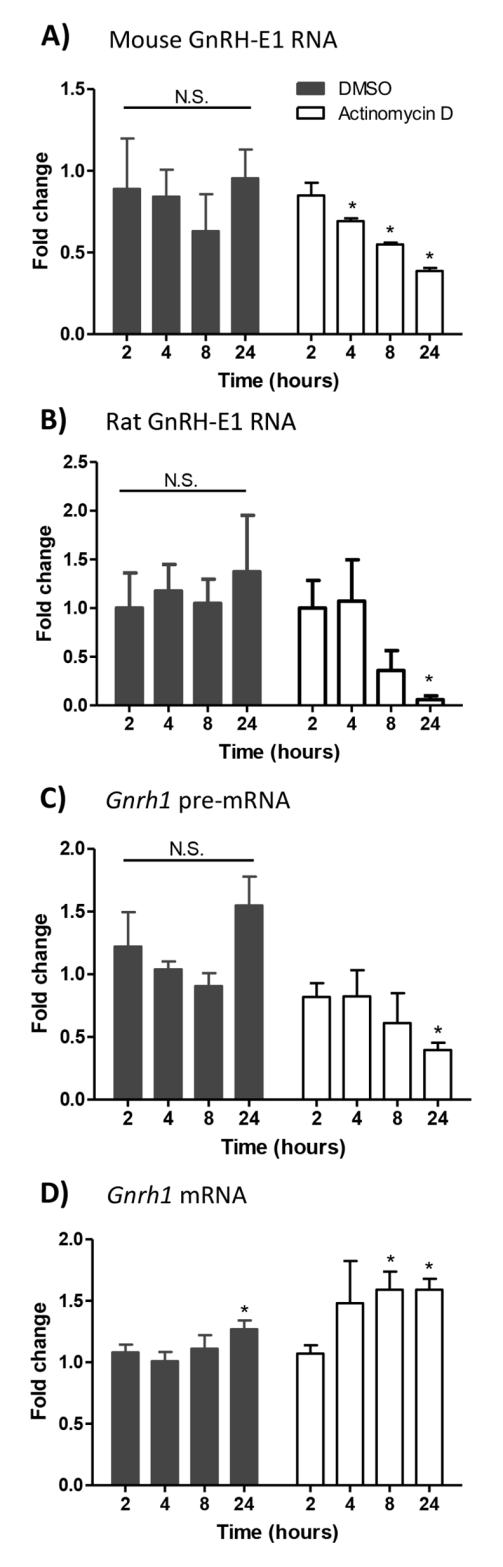
GnRH-E1 RNA, *Gnrh1* mRNA, and *Gnrh1* pre-mRNA stability following actinomycin D treatment of GT1-7 neurons. GT1-7 neurons were treated with either DMSO control vehicle (black bars) or 1 μg/mL actinomycin D (white bars). Total RNA was harvest at 2, 4, 8, and 24 hours after treatment. RT-qPCR analysis was performed to determine changes in endogenous mouse GnRH-E1 RNA (A), transgene-derived rat GnRH-E1 RNA (B), *Gnrh1* pre-mRNA (C) and *Gnrh1* mRNA (D) expression. Relative expression is normalized to *H2afz* mRNA control. Data are displayed as the fold change from untreated cells that were harvested at the time of treatment, and as the mean ± SD. Statistical significance was determined by two-way ANOVA, followed by *post hoc* Tukey-Kramer HSD, where asterisks indicate statistical significance at p<0.05.

LncRNAs are stable RNA molecules susceptible to siRNA knockdown and respond to cell signaling cascades [39, 40]. In contrast, the enhancer-derived noncoding RNAs (enhancer RNAs or eRNAs) can be short-lived, with half-lives of minutes, though they also respond to cell signaling cascades [41]. Our initial characterization of GnRH-E1 RNA showed that RNA expression from the *Gnrh1* gene regulatory region decreases in response to activation of the protein kinase C signaling pathway following TPA treatment [24]. Here, we examined the stability of GnRH-E1 RNA, compared to *Gnrh1* mRNA and *Gnrh1* primary transcript (pre-mRNA). GT1-7 neurons were treated with 1 μg/mL of actinomycin D, a transcription inhibitor, or DMSO control. Total RNA was harvested from 2 hours up to 24 hours following treatment (Fig 5). RT-qPCR analysis revealed a decline in mouse GnRH-E1 RNA abundance in actinomycin D-treated cells, with a 50% decrease in abundance between 8 hours and 24 hours after treatment (Fig 5A). Rat GnRH-E1 RNA showed a similar decline in abundance within 24 hours following actinomycin D treatment. *Gnrh1* pre-mRNA showed a similar rate of decrease in abundance after treatment (Fig 5C). *Gnrh1* pre-mRNA is likely continuously processed into mature *Gnrh1* mRNA. In contrast, *Gnrh1* mRNA was stable beyond 24 hours (Fig 5D). DMSO-treated cells showed similar expression levels of mouse and rat GnRH-E1 RNA, *Gnrh1* mRNA, and *Gnrh1* pre-mRNA up to 24 hours after treatment.

### Knockdown of Endogenous GnRH-E1 RNA Down-Regulates *Gnrh1* Gene Expression

**Fig 6:**
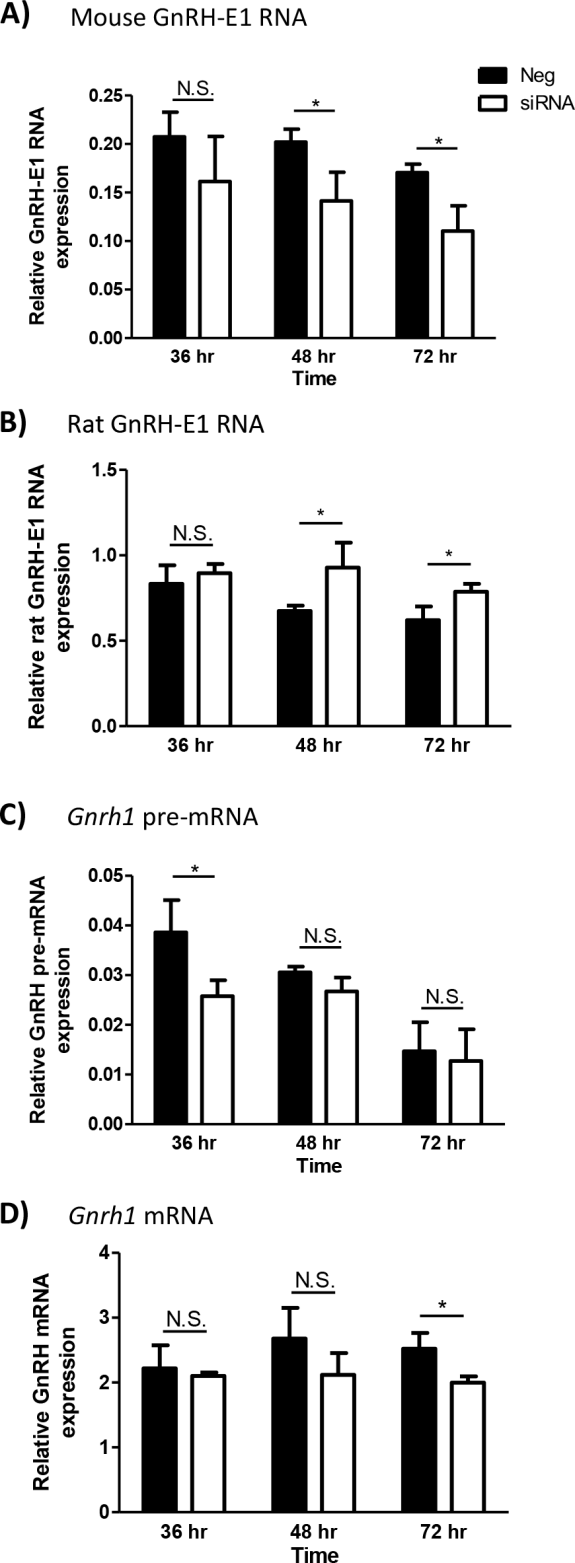
The effect of GnRH-E1 RNA knockdown on *Gnrh1* gene expression. (A-D) GT1-7 neurons were transfected with either negative siRNA control (Neg; black bars) or siRNA targeting both strands of the mouse GnRH-E1 RNA (siRNA; white bars). Total RNA was harvested at 36 hours, 48 hours, and 72 hours after siRNA transfection. RT-qPCR analysis was performed to quantify endogenous mouse GnRH-E1 RNA (A), transgene-derived rat (rTg) GnRH-E1 RNA (B), *Gnrh1* pre-mRNA (C), and *Gnrh1* mRNA (D) expression. Relative RNA expression is normalized to control histone 2A.Z (*H2afz*) mRNA expression at each time point. Data are displayed as the mean ± SD, where statistical significance was determined by Student’s t-test compared between negative control and siRNA treatment at each time point. Asterisk indicates statistical significance, where p<0.05.

To determine whether GnRH-E1 RNA functions in the regulation of *Gnrh1* gene transcription, siRNA knockdown of the endogenous mouse GnRH-E1 RNA was performed in GT1-7 neurons, followed by RT-qPCR assay of *Gnrh1* mRNA and *Gnrh1* pre-mRNA expression. We utilized a custom-designed siRNA duplex targeting both the sense and antisense variants of the endogenous mouse GnRH-E1 RNA (though not the E1 RNA expressed from the rat transgene). At 36 hours after siRNA transfection, we observed a small, not statistically significant, decrease in endogenous mouse GnRH-E1 RNA expression, compared to cells treated with negative control siRNA. Mouse GnRH-E1 RNA knockdown was more robust at 48 hours and 72 hours after siRNA transfection (Fig 6A). Rat GnRH-E1 RNA expression levels showed an increase at 48 hours and at 72 hours after siRNA treatment, compared to negative controls (Fig 6B), which indicate the specificity of siRNA targeting of the mouse GnRH-E1 RNA. Despite only a modest decrease in GnRH-E1 RNA at 36 hours after siRNA transfection, *Gnrh1* pre-mRNA expression was significantly reduced at 36 hours in cells treated with siRNA targeting GnRH-E1 RNA, compared to cells treated with negative siRNA control. *Gnrh1* pre-mRNA levels were not statistically affected by siRNA knockdown of GnRH-E1 RNA at 48 hours and 72 hours (Fig 6C). Importantly, *Gnrh1* mRNA expression showed a statistically significant decrease at 72 hours after GnRH-E1 RNA knockdown (Fig 6D), compared to negative control-treated cells. However, *Gnrh1* mRNA levels were not different between siRNA-treated cells and control-treated cells at 36 hours and 48 hours after siRNA transfection. *Gnrh1* mRNA levels appear to be maintained up to 37 hours after siRNA treatment, which may be due to the persistent effects of rat GnRH-E1 RNA in maintaining *Gnrh1* gene expression and/or the stability of *Gnrh1* mRNA. The effect of GnRH-E1 RNA knockdown on *Gnrh1* pre-mRNA suggests that GnRH-E1 RNA is functionally significant in the regulation of *Gnrh1* expression at the level of transcription.

### Overexpression of GnRH-E1 RNA Induces *Gnrh1* Transcriptional Activity in GN11 Neurons

**Fig 7:**
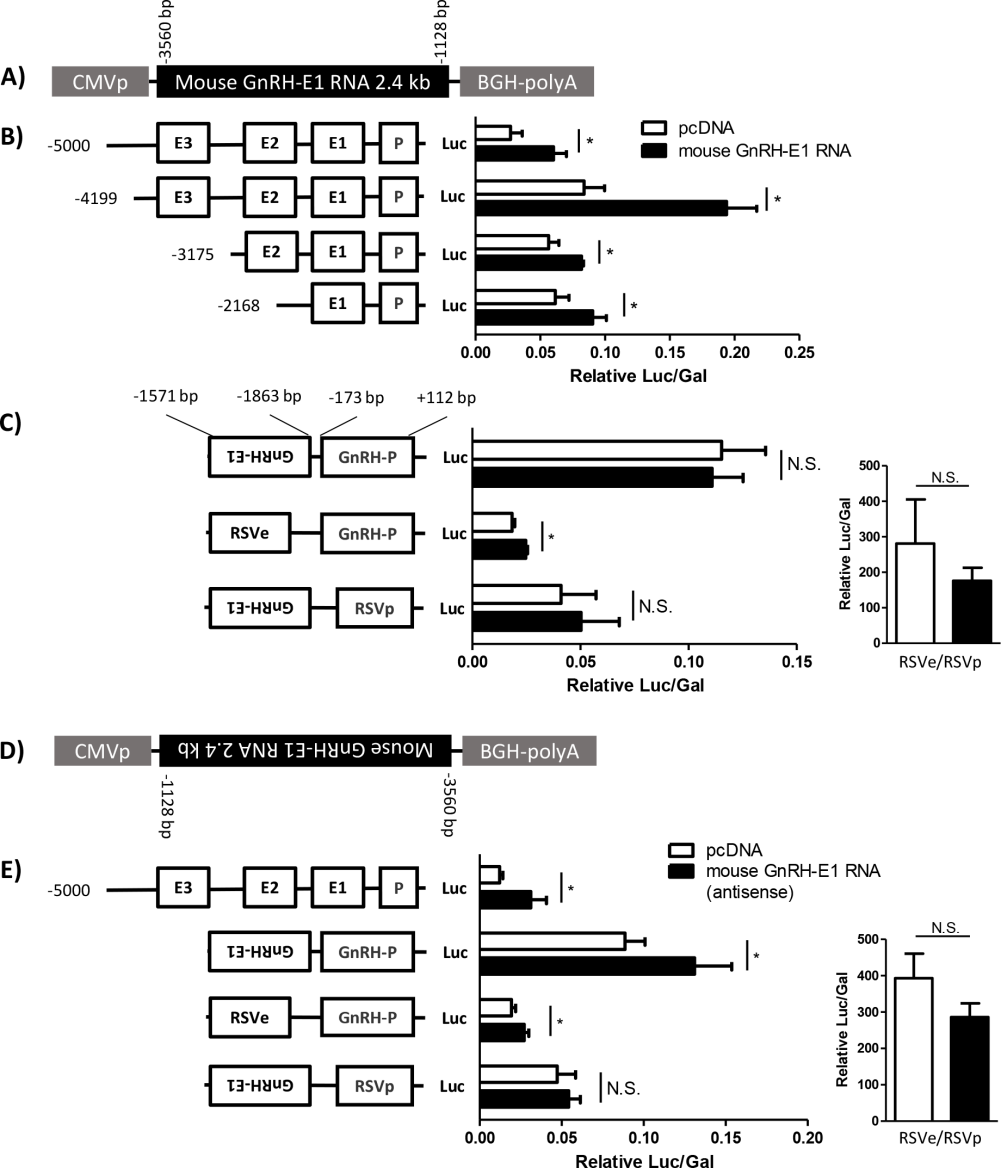
The effect of mouse GnRH-E1 RNA over-expression on *Gnrh1* gene transcriptional activity in GN11 cells. (A) A schematic diagram of the expression plasmid carrying the -3560 bp/-1128 bp segment from upstream of the mouse *Gnrh1* TSS, which contains the full-length mouse GnRH-E1 RNA that is integrated in the forward orientation. Expression of the sense GnRH-E1 RNA is driven by the CMV promoter (CMVp) and terminated by a plasmid-specific 3’ polyadenylation element (BGH-PolyA). B-C) GN11 neurons were transiently co-transfected with either empty pcDNA2.1 vector (white bars) or expression plasmid carrying the forward mouse GnRH-E1 RNA (black bars) for over-expression and the luciferase reporter plasmids as indicated. (B) GN11 cells were co-transfected with luciferase reporter plasmids containing -5000 bp, -4199 bp, -3175 bp, or -2168 bp of the rat *Gnrh1* regulatory region. Each luciferase reporter plasmid contains the indicated *Gnrh1* enhancers (E1, E2 and E3) and *Gnrh1* promoter (P). (C) GN11 cells were co-transfected with luciferase plasmids carrying the rat *Gnrh1* enhancer 1 and promoter (GnRH-E1/GnRH-P), RSV enhancer and the rat *Gnrh1* promoter (RSVe/GnRH-P), the rat *Gnrh1* enhancer 1 and RSV promoter (GnRH-E1/RSVp), or the RSV enhancer and promoter (RSVe/RSVp) control (inset). For reporter plasmids carrying GnRH-E1, the rat *Gnrh1* enhancer is integrated in the reverse orientation in the plasmid. (D) A schematic diagram of the antisense GnRH-E1 RNA expression plasmid carrying the full-length mouse GnRH-E1 RNA that is integrated in the reverse orientation, driven by CMVp and terminated by BGH-PolyA. (E) The antisense GnRH-E1 RNA expression plasmid (black bars) or empty pcDNA2.1 expression plasmid (white bars) were transiently co-transfected with a reporter plasmid carrying the -5 kb rat *Gnrh1* gene regulatory region, GnRH-E1/GnRH-P, GnRH-E1/RSVp, or RSVe/RSVp control (inset). Since the RSVe/RSVp is a very strong reporter, relative luciferase/β-galactosidase values for the RSVe/RSVp reporter are graphed separately for clarity in C and D. Luciferase/β-galactosidase values were normalized to pGL3. Data are displayed as the mean ± SD. Asterisks indicate statistical significance determined using Student’s T-test comparison between pcDNA2.1-transfected and GnRH-E1 RNA-transfected cells, where p<0.05.

The robust correlation between GnRH-E1 RNA and *Gnrh1* mRNA expression in GT1-7 neurons, as well as the marked decrease in *Gnrh1* gene expression after GnRH-E1 RNA knockdown in GT1-7 neurons suggest that GnRH-E1 RNA is a facilitator of *Gnrh1* gene transcription. We hypothesized that over-expression of GnRH-E1 RNA in GN11 neurons, where *Gnrh1* gene transcription is very low, could activate *Gnrh1* gene transcriptional activity. We constructed an expression plasmid carrying a genomic segment from -3560 bp to -1128 bp upstream of the mouse *Gnrh1* TSS, using a segment that contains the full-length GnRH-E1 RNA, integrated in the forward orientation in pcDNA2.1 expression plasmid. Expression of the sense GnRH-E1 RNA from the plasmid is driven by a constitutive heterologous promoter and terminated by a heterologous 3’ polyadenylation element (Fig 7A). In GN11 cells in culture, we transiently co-transfected the mouse sense GnRH-E1 RNA expression plasmid and luciferase reporter plasmids containing -5000 bp of the rat *Gnrh1* regulatory region and truncated reporter constructs containing -4199 bp, -3175 bp, and -2168 bp of the rat *Gnrh1* regulatory region. Over-expression of the mouse GnRH-E1 RNA induced transcriptional activity of the co-transfected *Gnrh1* gene regulatory elements, including -5000 bp, -4199 bp, -3175 bp, and -2168 bp of the rat *Gnrh1* gene regulatory region (Fig 7B).

We also asked whether the over-expression of the mouse sense GnRH-E1 RNA can activate the transcriptional activity of the *Gnrh1* enhancer and/or the promoter. In GN11 cells, we transfected luciferase reporter plasmids carrying the rat *Gnrh1* enhancer 1 and promoter (GnRH-E1/GnRH-P), the heterologous RSV enhancer and the rat *Gnrh1* promoter (RSVe/GnRH-P), the rat *Gnrh1* enhancer 1 and the heterologous RSV promoter (GnRH-E1/RSVp), or RSVe/RSVp control. The over-expression of the mouse sense GnRH-E1 RNA induced the activity of the *Gnrh1* promoter appended to RSVe, but did not affect the activity of GnRH enhancer 1 appended to RSVp or the activity of the reporter plasmid carrying GnRH-E1 and GnRH-P alone. Over-expression of the mouse sense GnRH-E1 RNA did not affect the activity of the control luciferase plasmid carrying RSVe/RSVp, compared to cells that were transfected with empty pcDNA2.1 control expression plasmid (Fig 7C).

We then tested whether the mouse antisense GnRH-E1 RNA also functions as a facilitator of *Gnrh1* transcriptional activity using an expression plasmid carrying the mouse GnRH-E1 RNA that is inserted in the reverse orientation, resulting in the CMV promoter-driving expression of the antisense GnRH-E1 RNA (Fig 7D). Over-expression of the mouse antisense GnRH-E1 RNA induced the transcriptional activity of -5000 bp of the rat *Gnrh1* regulatory region, the transcriptional activity of the rat GnRH-E1 and GnRH-P alone, and the activity of RSVe/GnRH-P, but did not affect the activity of the GnRH-E1/RSVp reporter. The over-expression of the mouse antisense GnRH-E1 RNA did not affect the activity of the RSVe/RSVp luciferase reporter plasmid, compared to cells that were transfected with empty pcDNA2.1 control expression plasmid (Fig 7E).

Together, these results indicate that both the sense and antisense variants of the mouse GnRH-E1 RNA can induce the transcriptional activity of co-transfected rat *Gnrh1* regulatory elements in immature GnRH neurons, where the activity of *Gnrh1* regulatory elements is normally silent. The observation that either the sense or antisense GnRH-E1 RNA can induce the activity of the rat *Gnrh1* promoter, appended to a heterologous RSV enhancer suggests that GnRH-E1 RNA acts to induce *Gnrh1* transcriptional activity specifically by affecting *Gnrh1* promoter activity.

## Discussion

Gonadotropin-releasing hormone gene expression and hormone secretion are highly specific functions of GnRH neurons in the hypothalamus. *Gnrh1* gene expression requires dynamic and specialized regulation by a number of molecular mechanisms. Previous studies in our laboratory first identified the expression of a *Gnrh1* enhancer-derived noncoding RNA (GnRH-E1 RNA) in the intergenic region of mouse *Gnrh1* and *Kctd9* by RT-PCR [24]. Here, we characterize the mouse GnRH-E1 RNA as a 5’ capped, 3’ polyadenylated, long intergenic noncoding RNA of over 2 kb in length, with variants transcribed in the sense and antisense direction in the upstream regulatory region of the *Gnrh1* that appear not to be spliced. Expression of GnRH-E1 RNA correlates with *Gnrh1* mRNA expression in a mature GnRH neuron-specific manner. Our data indicate that GnRH-E1 RNA functions as an inducer of *Gnrh1* gene transcriptional activity.

Our study is the first to describe the physical and functional characteristics of the mouse GnRH-E1 RNA, following our initial identification by RT-PCR and despite the absence of annotated RNA expression in publically available mouse or rat genome databases. However, human lincRNA RP11-395I14.2 (ENSG00000253476) has been identified and is transcribed from the intergenic region of human *GNRH1* and *KCTD9* in the same direction as *GNRH1* gene. Human lincRNA RP11-395I14.2 is 528 nt in length, is spliced, and contains a region in its exon 2 that is homologous to mouse GnRH-E1 RNA, but its function has yet to be described. While the human lincRNA RP11-395I14.2 expression profile is not exclusive to the brain, GnRH-E1 RNA expression in model cell lines, in contrast, suggests a hypothalamic cell-type specific expression pattern. Further investigation is required to examine whether human lincRNA RP11-395I14.2 and mouse GnRH-E1 RNA are functionally similar.

Strand-specific RT-PCR revealed both sense and antisense variants of the mouse GnRH-E1 RNA from rather discreet 5’ and 3’ ends located upstream and downstream of GnRH-E1. Although alternate transcription start and termination sites were indicated by RACE analysis, which also suggested expression of GnRH-E1 RNA variants of different lengths, strand-specific RT-PCR analysis revealed GnRH-E1 RNA variants of over 2 kb in length. Our amplification of shorter GnRH-E1 RNA variants within this 2 kb region from -3606 bp to -1128 bp upstream of the *Gnrh1* TSS using conventional PCR consistently produced full-size amplicons, in parallel with full-size products from genomic DNA positive control template. This observation suggests that GnRH-E1 RNA variants do not undergo splicing within the region between -3606 bp and -1128 bp. The sense GnRH-E1 RNA variant most likely contains a distinct 3’ end between -1443 bp and -1128 bp upstream of the *Gnrh1* TSS. The 5’ start site for GnRH-E1 RNA sense variant overlaps with the 3’ UTR of the *Kctd9* gene located upstream of *Gnrh1*. From our RNA sequencing analysis, a small decrease in RNA reads is observed that aligns to the center of GnRH-E1, but our strand-specific RT-PCR experiments showed that unlike small enhancer RNAs (eRNAs) [23, 41–43], the 5’ transcription start sites of the sense and antisense GnRH-E1 RNA variants do not originate from a central region of the enhancer. The 5’ transcription start sites reside in two separate locations at the 5’ region of GnRH-E2 and downstream of GnRH-E1.

Although alternate transcription start and termination sites were indicated by preliminary RACE analysis, which also suggested expression of GnRH-E1 RNA variants of different lengths, strand-specific RT-PCR analysis revealed GnRH-E1 RNA variants of over 2 kb in length. Our amplification of shorter GnRH-E1 RNA variants within this 2 kb region from -3606 bp to -1128 bp upstream of the *Gnrh1* TSS using conventional PCR consistently produced full-size amplicons, in parallel with full-size products from genomic DNA positive control template. Thus, we believe that at least some variants of GnRH-E1 RNA do not undergo splicing within the region between -3606 bp and -1128 bp. However, preliminary 5’ RACE analysis revealed capping structures located at approximately -3458 bp and at -1576 bp from the *Gnrh1* TSS. Preliminary 3’ RACE analysis revealed 3’ polyadenylation sites at -3720 bp and at -1182 bp from the *Gnrh1* TSS. It is possible that 5’ capping structures and 3’ polyadenylation sites predicted by RACE represent shorter GnRH-E1 RNA variants. On the other hand, the limitations of RACE analysis include 1) the limited ability to efficiently amplify long PCR products, 2) the efficiency of synthesizing cDNA from minor noncoding RNA variants for 5’ RACE, and 3) the identification of 5’ capping site and 3’ polyadenylation sites is heavily dependent on the location of gene-specific primers used for amplification and thus generates a bias toward expected ends. Predictions by RACE analysis may also reveal major variants, rather than all variants. Because of these limitations, we cannot rule out the possibility that the 5’ capping structures predicted by RACE represent shorter GnRH-E1 RNA variants, or that the 5’ ends identified by strand-specific RT-PCR analysis represent variants that were not identified by RACE. Nevertheless, cDNA synthesis using oligo-dT primers for RT-PCR and preliminary 3’ RACE analysis provides strong evidence that GnRH-E1 RNA variants are polyadenylated. Together, the physical characteristics of GnRH-E1 RNA are consistent with those of lncRNA and lincRNA, rather than eRNA.

Furthermore, the stability of GnRH-E1 RNA is consistent with the characteristics of lncRNA, rather than eRNA. LncRNAs can be stable RNA molecules that are susceptible to transient knockdown by siRNA and provide sustained regulation of gene expression [40]. In contrast, eRNAs are rather short lived, with half-lives of minutes [41]. The stability of *Gnrh1* mRNA is evidently maintained for more than 24 hours, whereas the stabilities of GnRH-E1 RNA and *Gnrh1* pre-mRNA are comparatively shorter, with half-lives of about 8 hours. Nevertheless, we predicted that GnRH-E1 RNA is sufficiently stable for transient siRNA knockdown. In addition, the observation that GnRH-E1 RNA resides in the nucleus provides an important insight into the function of GnRH-E1 RNA as potential regulator of *Gnrh1* gene expression at the level of transcription.

For siRNA knockdown of GnRH-E1 RNA, we utilized a custom-designed siRNA duplex targeting a region that is common in both the sense and antisense mouse GnRH-E1 RNA. A significant knockdown of endogenous mouse GnRH-E1 RNA was observed in GT1-7 neurons, accompanied by a decrease in *Gnrh1* pre-mRNA at 36 hours and a decrease in *Gnrh1* mRNA at 72 hours after siRNA treatment. The delayed decrease in *Gnrh1* mRNA levels may reflect the long half-life (> 24 hours) and stability of *Gnrh1* mRNA (Fig 6D). It is also important to note that our siRNA specifically targets only the mouse GnRH-E1 RNA, and does not target the rat GnRH-E1 RNA expressed from the rat transgene. RT-qPCR analysis indicated that transgene-derived rat GnRH-E1 RNA expression was maintained at 36 hours after siRNA transfection. The expression of rat GnRH-E1 RNA was higher in siRNA-treated cells, compared to control-treated cells, at 48 hours and 72 hours. Future studies on the mechanisms of GnRH-E1 RNA action may provide insight into the possible causes and mechanisms of the apparent increase in rat GnRH-E1 RNA expression while the endogenous mouse GnRH-E1 RNA is lowered after siRNA treatment. Nevertheless, the rat GnRH-E1 RNA is likely acting to maintain *Gnrh1* expression during the siRNA knockdown of the mouse GnRH-E1 RNA. Even so, the knockdown of only the mouse GnRH-E1 RNA caused a down-regulation of both *Gnrh1* mRNA and the *Gnrh1* primary transcript. These observations provide evidence that GnRH-E1 RNA is a facilitator of *Gnrh1* gene expression.

*Gnrh1* gene expression is tightly controlled by numerous mechanisms, including chromatin modifications and the action of transcription factors which act on the *Gnrh1* upstream regulatory elements, notably *Gnrh1* enhancers and promoter. The effect of GnRH-E1 RNA knockdown on *Gnrh1* mRNA and *Gnrh1* pre-mRNA in GT1-7 cells may be a reflection of the various molecular mechanisms that tightly control *Gnrh1* gene expression in addition to GnRH-E1 RNA. A number of transcription factors and homeodomain proteins have been shown to bind at the rat *Gnrh1* enhancer region. OCT1, a POU homeodomain protein, and TALE homeodomains PREP1/PBX binding at the *Gnrh1* regulatory region is observed in GT1-7 cells, but not GN11 and NIH3T3 cells, suggesting neuron-specific control of *Gnrh1* expression [44–46]. The zinc finger protein GATA-4 was also shown to bind to the rat *Gnrh1* regulatory region (-1571 to -1863) [47, 48] during GnRH neuron migration in development, but is not found in adulthood [49], which suggests temporal regulation of *Gnrh1* expression. In addition, Q50 non-Hox homeodomain transcription factors MSX and DLX bind directly to CAATTA repeat elements in both GnRH-E1 and promoter. In GT1-7 cells, co-transfection of MSX1 or 2 and reporter plasmid carrying *Gnrh1* enhancer and promoter showed repression of *Gnrh1* promoter activity, while DLX2 or 5 relieved the repression, suggesting dynamic control of enhancer and promoter activity. In fact, mice lacking MSX or DLX showed abnormal numbers and spatial distribution of GnRH neurons throughout development, suggesting critical role of these transcription factors at the *Gnrh1* regulatory region [33, 50]. Two other transcription factors occupy the ATTA repeat elements in both GnRH-E1 and the promoter: homeodomain transcription factors sine oculis-related homeobox (SIX6) and ventral anterior homeobox 1 (VAX1), both of which act as inducers *Gnrh1* expression and are highly expressed specifically in GT1-7 neurons. Mice lacking SIX6 present with dramatically decreased hypothalamic GnRH neuron number, disrupted reproductive development, and severely reduced reproductive capacity [51]. The absence of VAX1 in GnRH neurons results in infertility and the absence of hypothalamic GnRH neurons, observed as early as late embryonic development. Both SIX6 and VAX1 are required for the maintenance of *Gnrh1* expression during GnRH neuron maturation in embryonic development. Interestingly, VAX1 is a relatively modest activator of *Gnrh1* transcription, but competes with SIX6, a strong activator of *Gnrh1* transcription, for occupation at the ATTA repeat element [52].

One possible mechanism of GnRH-E1 RNA function is through the interaction with transcription factors that are known to control *Gnrh1* expression. The decrease in GnRH-E1 RNA may lower the efficiency of transcription factor binding at *Gnrh1* regulatory elements, but compensatory mechanisms and the other transcription factors may also play a role in maintaining *Gnrh1* transcription. In addition, GnRH-E1 RNA can act in *trans*, as evidenced by the effect of GnRH-E1 RNA overexpression on transfected *Gnrh1* regulatory elements. GnRH-E1 RNA may also act on distal genes, such as key transcription factors that regulate *Gnrh1* gene transcription, and thus acting indirectly. In GT1-7 neurons, where *Gnrh1* gene expression is robust and controlled by numerous factors, GnRH-E1 RNA action may play only a limited role in facilitating the transcription of *Gnrh1* gene. Nevertheless, our data showed that the knockdown of the mouse GnRH-E1 RNA alone results in a substantial decrease in endogenous *Gnrh1* gene expression.

Furthermore, the strength of GnRH-E1 RNA regulation of *Gnrh1* gene expression we observed in our siRNA knockdown experiment is rather comparable to the vigor of lncRNA effects on individual target gene expression. Even among well-characterized lncRNAs described in the literature, the depletion of a lncRNA can generally down-regulate or remove repression of individual target gene expression [38, 53–55], but by and large does not completely abolish target gene expression. Even so, in these studies, changes lncRNA expression and the effect on their respective target gene(s) have been associated with changes in cell function, cell lineage commitment, and changes in cell differentiation states. In animal studies of lncRNA function, changes in the expression of a lncRNA can amplify into changes in physiological states and pathology of disease [35, 37, 40, 56].

To firmly establish the function of GnRH-E1 RNA as an inducer of *Gnrh1* gene expression, we tested the effect of the mouse sense and antisense GnRH-E1 RNA on the transcriptional activity of extensively studied and well-characterized rat *Gnrh1* regulatory elements [15–17, 33]. Indeed, the over-expression of either the sense or antisense mouse GnRH-E1 RNA in GN11 cells resulted in a substantial, up to 2-fold, increase in the transcriptional activity of co-transfected rat *Gnrh1* regulatory elements. This observation not only demonstrates the high level of evolutionary conservation between the mouse and rat *Gnrh1* regulatory region, but is also consistent with our characterization of GnRH-E1 RNA transcribed in both the sense and antisense direction. Importantly, the data indicate that GnRH-E1 RNA may indeed act as an inducer of *Gnrh1* gene transcription, and that both sense and antisense strands are functionally significant. GN11 neurons represent immature and migratory GnRH neurons, where *Gnrh1* mRNA is absent, and GnRH-E1 RNA expression is not detected. The transcriptional activity of transiently transfected *Gnrh1* regulatory elements is relatively silent in GN11 neurons, compared to the activity in GT1-7 neurons. The transcriptional activity of *Gnrh1* in GT1-7 neurons can be 100–1000 times higher than that in GN11 neurons, as shown in the difference in RNA detection between GT1-7 cells and GN11 cells by RNA sequencing. Nevertheless, over-expression of GnRH-E1 RNA resulted in a marked increase in the transcriptional activity of *Gnrh1* regulatory elements. The data indicate that GnRH-E1 RNA is an inducer of *Gnrh1* gene transcription, thus may play a role in activating or de-repressing the transcriptional activity of *Gnrh1* gene regulatory elements in differentiating GnRH neurons.

Furthermore, we determined that the positive effect of GnRH-E1 RNA on *Gnrh1* regulatory elements requires the *Gnrh1* promoter, suggesting that GnRH-E1 RNA induction of *Gnrh1* transcription is through the induction of *Gnrh1* promoter activity. Interestingly, the mouse sense GnRH-E1 RNA induced the activity of the reporter plasmid containing the -2168 bp rat *Gnrh1* regulatory region which includes the enhancer and promoter, but did not affect the activity of the GnRH-E1/GnRH-P reporter. This observation may indicate that action of the sense GnRH-E1 RNA requires the genomic region between the enhancer and the promoter. On the other hand, the antisense GnRH-E1 RNA induced the activity of GnRH-E1 and GnRH-P, as well as GnRH-P with an RSV enhancer, which provides strong indication that the GnRH-E1 RNA induces *Gnrh1* gene expression by inducing the activity of the *Gnrh1* promoter.

In this study, we established the mouse *Gnrh1* enhancer-derived noncoding RNA, GnRH-E1 RNA, as a facilitator of *Gnrh1* gene transcription in GnRH neuronal cell lines. Sense and antisense variants of GnRH-E1 RNA are 5’ capped and 3’ polyadenylated RNA molecules transcribed from the *Gnrh1* upstream regulatory region, but transcription evidently does not originate from a central region. The physical characteristics of GnRH-E1 RNA are consistent with those of lincRNAs. Our data indicate that GnRH-E1 RNA functions as an inducer of *Gnrh1* transcription by inducing the activity of the *Gnrh1* promoter, and may play a critical role in activating or de-repressing *Gnrh1* transcription during GnRH neuron maturation and development. Together, these data provide a foundation for future studies on the role of lincRNA in GnRH neuron function and maturation, with implications in reproductive neuroendocrinology.
